# Facebook Groups on Chronic Obstructive Pulmonary Disease: Social Media Content Analysis

**DOI:** 10.3390/ijerph16203789

**Published:** 2019-10-09

**Authors:** Avery Apperson, Michael Stellefson, Samantha R. Paige, Beth H. Chaney, J. Don Chaney, Min Qi Wang, Arjun Mohan

**Affiliations:** 1 Department of Health Education and Promotion, East Carolina University, Greenville, NC 27858, USA; 2 STEM Translational Communication Center, University of Florida, Gainesville, FL 679205, USA; 3 Department of Behavioral and Community Health, University of Maryland, College Park, MD 20742, USA; 4 Division of Pulmonary, Critical Care and Sleep Medicine, Department of Internal Medicine, East Carolina University Brody School of Medicine, Greenville, NC 27858, USA

**Keywords:** COPD, Facebook, social media, online community, self-management, social support

## Abstract

Facebook Groups facilitate information exchange and engagement for patients with chronic conditions, including those living with Chronic Obstructive Pulmonary Disease (COPD); however, little is known about how knowledge is diffused throughout these communities. This study aimed to evaluate the content that is available on COPD-related Facebook Groups, as well as the communication (self-disclosures, social support) and engagement (agreement, emotional reaction) strategies used by members to facilitate these resources. Two researchers independently searched the “Groups” category using the terms “COPD”, “emphysema”, and “chronic bronchitis”. Twenty-six closed (*n* = 23) and public (*n* = 3) COPD Facebook Groups were identified with 87,082 total members. The vast majority of Group members belonged to closed (*n* = 84,684; 97.25%) as compared to open (*n* = 2398; 2.75%) groups. Medications were the most commonly addressed self-management topic (*n* = 48; 26.7%). While overall engagement with wall posts was low, the number of “likes” (an indicator of agreement) was significantly greater for wall posts that demonstrated social support as compared to posts that did not (*p* < 0.001). Findings from this study showed that COPD Facebook group members share specific disease-related experiences and request information about select self-management topics. This information can be used to improve the quality of self-management support provided to members of popular COPD Facebook groups.

## 1. Introduction

Chronic Obstructive Pulmonary Disease (COPD) refers to a group of respiratory diseases, including chronic bronchitis and emphysema, characterized by airflow blockage and a progressive worsening of breathing [1]. Airflow limitation is caused by thickening and inflammation of the airway lining and destruction of the tissues that allow for gas exchange to occur. As COPD progresses, symptoms often worsen and become disabling during the everyday lives of people living with the condition [2]. COPD is currently the fourth leading cause of death in the United States [3]. This debilitating chronic condition is also responsible for killing more than three million people worldwide every year [4]. There are currently 16 million people diagnosed with COPD in the United States (U.S.) [5], and approximately 12 million adults are likely living with COPD but remain undiagnosed [6]. COPD is commonly underdiagnosed in people who are not current or past smokers and young adults who have minimal breathing limitations [7]. By 2030, the World Health Organization (WHO) anticipates that COPD will become the third leading cause of mortality and seventh leading cause of morbidity worldwide [8].

In the U.S., annual healthcare expenses attributable to COPD and its sequelae cost is in excess of $32 billion dollars. However, these costs could be even higher due to the large amount of people likely to be living with COPD who remain undiagnosed. Medical costs associated with COPD are projected to approach $49 billion dollars by 2020 [9]. An estimated 16.4 million days of missed work are caused by COPD, causing annual indirect costs associated with lost productivity due to COPD to be approximately $3.9 billion dollars [10]. As annual healthcare expenditures attributable to COPD continue to rise, secondary and tertiary prevention, including screening for COPD via spirometry and successful smoking cessation will be increasingly important to help manage medical costs related to COPD [10]. 

Symptoms of COPD often include wheezing, productive cough, shortness of breath, and chest tightness [5]. These symptoms often worsen over time, which can be disabling for many people diagnosed with the condition. A worsening, or flare up, of these symptoms is known as an exacerbation. An exacerbation of symptoms is usually due to an infection of the lungs or airways; however, sometimes the cause of an exacerbation is unknown. Inhaling irritating pollutants and allergies can sometimes be the cause of symptom exacerbations [11]. In 2008, there were 822,500 documented hospital stays attributed to COPD in the U.S. among people 40 years of age and older [12]. Hospitalization rates for acute exacerbations of COPD were higher in the Midwest and South, particularly in rural and low-income areas [12]. The average length of stay for a hospital visit was 4.8 days, and the average cost of a COPD-related hospital stay was $7500 [12]. 

Prolonged exposure to tobacco smoke is by far the most significant risk factor for COPD. Cigarette smoking produces many toxins that destroy lung tissues, cause inflammation in airways, and weaken the ability of the lungs to prevent infections [13]. Other risk factors for COPD include poor indoor air quality, second-hand smoke, exposure to toxins, older age, and genetic factors including Alpha-1 antitrypsin deficiency [14]. COPD is diagnosed based on symptoms, degree of airflow limitation, risk for exacerbations, and identifiable comorbidities. Common comorbidities associated with COPD include cardiovascular disease, diabetes, anemia, obstructive sleep apnea, gastroesophageal reflux disease, anxiety, and depression [15].

Although there is currently no cure for COPD, treatment can slow the progression and control symptoms [5]. Common types of medications used to treat COPD are bronchodilators, oral steroids, inhaled steroids, antibiotics, and combination inhalers. Bronchodilators, such as albuterol, work to relieve coughing and shortness of breath by relaxing the muscles around the airways. Inhaled steroids and oral steroids help prevent and relieve exacerbations by reducing airway inflammation, while combination inhalers use a mixture of inhaled steroids and bronchodilators. Respiratory infections can worsen COPD symptoms and are treated with antibiotics such as azithromycin [16]. Poor medication adherence among patients with COPD often results in adverse health outcomes, reduced quality of life, higher hospitalization rates, and increased healthcare expenditures [17]. 

While COPD treatments such as pulmonary rehabilitation, bronchodilators, nutritional counseling, and smoking cessation can alleviate symptoms and decrease exacerbations [1], many patients receive poor information on lifestyle changes and ways to manage their disease [18]. People living with chronic diseases are increasingly using the Internet to gain knowledge of specific diseases and their treatments [19]. Studies show that patients with COPD are moderately confident making health decisions based on information gained from the Internet [20]. However, patients with COPD are less confident in their ability to distinguish between low and high-quality sources of health information [21]. The introduction of technology into disease management has generated mixed perceptions from patients. Some see the benefits—such as early detection of exacerbations and awareness of symptoms—while others believe it creates a divide between the patient and health care professional [22].

Social media platforms provide an online space for Internet users to create user profiles, make connections with other users, and engage in online discussions [23]. Social media is used by 69% of Americans today. Although young adults continue to be the largest users of social media, older adults have become more accustomed to using social technologies. In 2016, 80% of 30- to 49-year old and 64% of 50- to 64-year old adults were using at least one type of social media. Among the major social media platforms, Facebook is the most widely used [24]. Sixty-eight percent of U.S. adults report using Facebook, with approximately 75% of these users visiting the site daily [25]. 

People with chronic diseases are now using online social networks to seek advice and obtain evidence-based health information to help with self-managing their conditions [19]. According to Allen, Vassilev, Kennedy and Roger [26], “social ties forged in online spaces provide the basis for performing relevant self-management work that can improve an individual’s illness experience, tackling aspects of self-management that are particularly difficult to meet offline.” (p. e61). Free social networking sites, such as micro-blogs (e.g., Twitter), Facebook, Pinterest, and discussion boards, allow users to share and obtain information through user-friendly platforms. In addition, these sites could be useful for reaching specific, intended audiences; for example, a social media content analysis of Pinterest found that this social networking website is highly marketed to women as a resource for living with COPD [27]. Therefore, the research suggests that the use of certain social media websites, in this case—Pinterest, may be useful for disseminating health information to specific audiences, thus allowing for audience segmentation. 

New media tools, like social networks, are also re-engineering the way doctors, patients, and their caregivers interact with each other. Results of a systematic review investigating social media use among health professionals suggest that health care providers perceive social media platforms to be useful tools in facilitating chronic disease self-management with patients [28]. Social networks allow greater accessibility to health-related information and provide an outlet for communication between people living with a similar chronic disease(s) [29]. Research shows that social media has been used for diagnostic purposes, patient education, and disease management [30]. Moreover, there has been an increase in the number of social media-based health management systems used to deliver more convenient health care services. Gu and colleagues [31] found that a patient’s personality (openness to new experiences) impacts their use of social media-based health management systems. However, research on the use of social networks for disease prevention and management is limited. Patel and colleagues [30], note that although it is evident technology has the potential to improve public health, it is necessary to conduct further research on how patients and technology interact to improve disease management and outcomes. 

Facebook is a popular online social network that allows users to share photos, posts, and engage in discussions [32]. With over one billion active daily users [33], Facebook has a broad reach for sharing information related to chronic disease. Sixty percent of state health departments report using social media, with fifty-six percent having a Facebook account [34]. When patients access health-related information on Facebook, their main motives are social support, exchange of advice, and increasing knowledge [35]. Facebook “Groups” are unique forums used by Facebook users who share common interests. These Groups allow Facebook users to communicate about a common organization, event, or issue in a variety of formats (e.g., photos, text) [36]. Content is communicated through self-disclosures and support-reciprocating topics and engaged in the form of agreements and contributions (e.g., comments) [36]. Disease-specific information exchanges now occur within Facebook Groups. 

Past studies have shown Facebook Groups to be a communication tool used by patients seeking information or support for breast cancer [37], depression [38], and diabetes [39]; however, there are no studies examining what self-management content is communicated on COPD-related Facebook groups and how the content is engaged among its members. Users communicate content through self-disclosures and support, whereas, this content is engaged through reactions (e.g., agreement and expression, comments). Exploring how Facebook Groups host COPD self-management information content will inform researchers and practitioners about what content is available and diffused within online COPD communities. To this end, three research questions (RQ) were proposed.

RQ1: What (a) self-management content areas, (b) communication strategies, and (c) engagement metrics are available on COPD Facebook Groups? 

RQ2: Are certain communication strategies more common on COPD Facebook Group wall posts that mention (a) generic self-management, (b) medication management, and (c) hospitalization/doctor visits?

RQ3: Which communication strategies yield most user engagement on COPD Facebook Group wall posts? 

## 2. Materials and Methods

### 2.1. Theoretical Framework 

Understanding the processes and variables involved in health behavior, lifestyle change, and self-management allows for better communication of health enhancing messages [40]. The Common-Sense Model of Illness Self-Regulation (CSM) examines the perceptual, behavioral, and cognitive responses that patients often have when self-managing their health threats [40]. The beliefs and expectations people have about an illness, or illness representations, is the key construct in this model [41]. These expectations inform the ways in which patients navigate their responses to the illness, including what decisions patients make for managing the illness. Major beliefs involved in illness representations of COPD include comorbid identities (i.e., asthma), causes (i.e., cigarette smoking), consequences (i.e., mental health disorders), and curability (i.e., rescue medications, pursed lipped breathing techniques). Relationships among these beliefs tend to guide how patients cope with and treat their own chronic illness(es). Social media is one mechanism for providing self-management support for coping and treating chronic illness(es) [30]; however, research on how concepts of CSM can be utilized on this platform for the promotion of successful self-management of chronic illness(es) is limited [40]. Therefore, a better understanding of how social media is being used to motivate and engage users, as a way to self-manage a health threat, and communicate content regarding chronic illness(es) is warranted.

### 2.2. Search Procedures

Two researchers individually searched the “Groups” category of Facebook using the terms “COPD”, “emphysema”, and “chronic bronchitis” to locate existing both public and closed COPD groups. Closed Facebook groups are defined as groups requiring approval from an administrator or current member to join before Group content can be viewed. Public groups are those that can be joined by any Facebook user with a valid account; any Facebook user can view group wall post content of public groups, even if they are not a member of group. Both public and closed Facebook groups allow all Facebook members to view the group title, description, members, and post activity, even if they are not a member of the group. For closed Facebook groups, administrators are contacted to gain access to the group’s wall post content. Closed groups commonly have questions that accompany a request to join, such as asking about the Facebook user’s reason for joining the group. These questions were answered by each member of the research team by responding in the following manner: “Please see message in private inbox before accepting this request. Thank you”. The message sent to each group administrator’s private inbox (Appendix A) was approved by East Carolina University’s Institutional Review Board (IRB).

### 2.3. Inclusion/Exclusion Criteria 

The analysis was restricted to all public and closed Facebook Groups related to COPD with content posted content in English. Secret Facebook groups, or those that did not show up in Facebook group searches, were excluded from the analysis because only individual Facebook users who have been invited by the Secret group administrator or a current Facebook member have the ability to see the group title, description, members, and wall post content. Any closed Group that did not accept the researchers’ request to join the Facebook Group by the beginning of data collection was excluded because wall posts can only be viewed by Facebook group members. However, we were able to record general membership data about closed groups, as this information is publicly accessible to anyone with a valid Facebook account. 

### 2.4. Data Collection 

**Figure 1:**
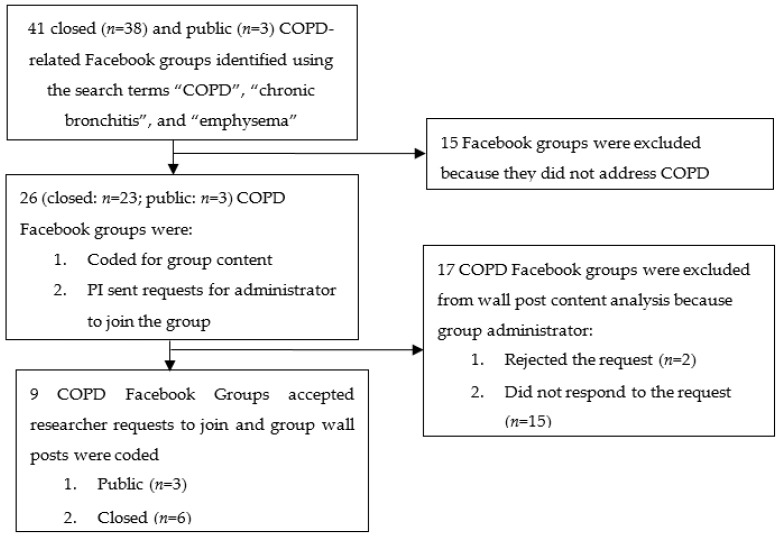
Flowchart depicting Chronic Obstructive Pulmonary Disease (COPD) Facebook group selection process.

Institutional Review Board (IRB) approval was obtained from the researchers’ university prior to collecting data from Facebook. Twenty-six closed (*n* = 23) and public (*n* = 3) COPD Facebook Groups were identified after accounting for inclusion and exclusion criteria as described in Figure 1. Fifteen Facebook groups were initially excluded (*n* = 15) because they focused on other pulmonary diseases and disorders other than COPD. After excluding these groups, 26 were retained for further analysis. Of these, 17 were excluded due to group administrators not responding to our request to join their group (*n* = 15) or disallowing our request to join (*n* = 2). The two COPD Facebook group administrators who declined investigator requests to join their group did not provide a specific reason for doing so. Upon accounting for exclusion criteria, researchers recorded membership statistics and coded wall posts from three public and six private COPD Facebook groups. 

Two researchers independently reviewed these nine COPD Facebook groups and coded publicly-accessible group information such as group title, number of members, and wall post activity. Member accessibility to Facebook Groups (closed or public) was noted by the researchers. Based on prior social media content analysis research [37,38,40] a coding and classification scheme was applied to the 20 most recent wall posts within nine closed (*n* = 6) and public (*n* = 3) Facebook COPD groups. This resulted in 180 unique wall posts considered for analysis. 

### 2.5. Measures 

Demographics of each COPD Facebook Group and wall post were examined. For the groups, post activity included the number of members gained or lost in the last 30 days, number of new daily posts, and number of posts made in the last 30 days. In addition, researchers recorded the privacy setting (closed or public), intended audience (patient, caregiver, health care provider), and number of months each COPD Facebook group was in existence. For COPD Facebook Group wall posts, reach (number of members in each group) and engagement (wall post activity) metrics were recorded. Group wall posts were also coded to determine the type of media modality (i.e., text, video, photo, and infographics). 

Table 1 shows the categories and definitions for each code in the analysis. Group titles and descriptions were coded to determine the purpose of the group related to the following categories: (1) building awareness/sharing information, (2) providing support, (3) fundraising purpose, (4) marketing/promoting a product or service, or (5) other. Wall posts were coded based on self-management content areas: (1) mental health, (2) medication management, (3) hospitalizations, (4) asthma, (5) cigarette smoking, (6) breathing techniques, (7) nutrition, and (8) physical activity. Communication strategies were recorded using the following categories: (1) self-disclosures about the disease; (2) referrals; (3) information-providing, (3) requests for information, (4) demonstrations of support, (5) product/service promotion, and (6) other (See Table 1 for definitions of group purpose codes). Engagement metrics such as number of reactions (i.e., likes, emojis for love, funny, surprise, sad, and anger), comments, and shares of wall posts were recorded. Use of emoticons or “emojis” to evaluate user reactions may not accurately reflect all user attitudes about Facebook content, but these visual communication tools can serve as a useful proxy for assessing user reactions to health care content found on social media [41].

### 2.6. Coder Training

A codebook was developed based on existing social media content analysis research [37,38,40] and relevant COPD self-management guidelines [44,45]. Two researchers met before data collection to discuss the coding methods operationalized in the codebook and resolved discrepancies in how codes were to be interpreted. Subsequently, each researcher independently extracted data from COPD Facebook groups and recorded the data. Intercoder reliability of applied codes was determined using Cohen’s Kappa Statistic [47]. The Kappa statistic was selected, because it serves as a robust measure of intercoder reliability in research that involves the coding of behavior-related variables [48], including those measured on social media. A subset of 10–25% of the total sample was sufficient for determining inter-rater reliability of codes; therefore, a sub-sample of 5 Facebook groups was independently coded by each researcher. The cut-off value for acceptable intercoder reliability was set at 0.70 [49].

### 2.7. Data Analysis 

The user analytic data was exported to Statistical Package for Social Sciences (SPSS) v24.0 for further analyses. Frequency and descriptive statistics were computed to determine the group purpose, membership levels, wall post activity, and group member engagement activity within COPD Facebook groups. Nonparametric data led to reporting medians (±IQR). Descriptive median (±IQR) statistics were used to determine average engagement (likes, comments, etc.) metrics for wall posts addressing various aspects of self-management (mental health, medication management, etc.). Chi-square analyses were conducted to determine if the number of wall post communication strategies (self-disclosure, referral, information providing, information requesting, offering support, and product/service promotion) varied based on whether or not (a) general self-management, (b) medication management or (c) hospitalizations were specifically addressed. A series of Mann-Whitney *U* tests were conducted to examine the extent to which wall post communication strategies (i.e., providing information, requesting information, and/or demonstrating support) were associated with group member engagement (i.e., number of wall post likes, sad emojis, and comments).

## 3. Results

### 3.1. Data Reliability 

Kappa agreement between coders ranged from 0.66–0.99 (M = 0.90, SD = 0.15) for the self-management content areas (Table 2). Kappa estimates for wall post characteristics ranged from 0.66–0.99 (M = 0.91, SD = 0.12). All Kappa statistics were at or near the cut-off value used to establish adequate intercoder reliability (0.70).

### 3.2. Group Characteristics 

The purpose of most COPD Facebook groups was to provide support (19/26, 73.1%), while the remaining groups (7/26, 26.9%) built awareness or shared health information. None of the groups had a primary purpose of fundraising or marketing/promoting a product or service. The median time in existence for both public and closed groups was 60 months (*IQR*= 30 months to 90 months), or about five years. There were no statistically significant differences in the median number of members, growth in members over the past 30 days, new daily posts, and number of wall posts in the past 30 days based on group purpose.

### 3.3. Group Size

Table 3 describes the general characteristics and membership statistics for the 26 COPD Facebook groups analyzed in this content analysis. In total, 87,082 members were identified as members of these groups. The vast majority of members belonged to closed (*n* = 84,684; 97.25%), as opposed to open (*n* = 2398; 2.75%) groups. The number of members ranged from 153 members in the smallest group (“Chronic Bronchitis Support & Awareness”) to 12,337 members in the largest group (“Ultimate Pulmonary Wellness: COPD, PF, Pulmonary Hypertension and Others”). The largest group was closed, had a group purpose of providing support, and was in operation for 36 months or approximately 3 years.

### 3.4. Wall Post Content and Characteristics 

The most common communication strategy used in wall posts was self-disclosure (80/180, 44.4%), followed by referrals to external web sources of information (53/180 or 29.4%). Forty-five (25%) wall posts provided information related to COPD, while 69 (38%) requested information about COPD. Wall posts that offered messages of support comprised 14.4% (26/180) of total group wall posts, while only 2.7% (5/180) of wall posts promoted a product or service. 

Specific self-management content areas most often included in group wall posts were medication management (48/180, 26.7%) and hospitalizations/doctor visits (28/180, 15.6%). Other generic aspects of self-management, including mental health (14/180, 7.8%), cigarette smoking (10/180, 5.6%), physical activity (8/180, 4.4%), asthma (5/180, 3.3%), breathing techniques (5/180, 2.8%), and nutrition (1/180, 0.6%), received less attention.

### 3.5. Group Wall Post Engagement 

There was low overall engagement on wall posts. The median number of wall post “likes” was four (*IQR* = 8.5), while the median number of comments was five (*IQR* = 12). Love emojis, sad emojis, angry emojis, “haha” emojis, wow emojis, and shares of wall posts were virtually non-existent (*Mdn* = 0, *IQR* = 0). 

### 3.6. Wall Post Communication Strategy and Self-Management 

Wall posts that addressed self-management were more likely to include a self-disclosure (62.5%), as compared to posts that did not address self-management (28%), *χ*^2^(*df* = 1) = 21.54, *p* < 0.001 (Table 4). A greater proportion of self-management posts posed questions to other members (60.9%), *χ*^2^(*df* = 1) = 14.01, *p* < 0.001, but a lesser proportion provided support to fellow group members (7.7%), *χ*^2^(df = 1) = 15.72, *p* < 0.001. These estimates are compared to self-management posts that did not request information (32.4%) but provided support (49.4%) to fellow group members. Self-management posts were also less likely to refer members to external web sources (24.5% vs. 51.2%), *χ*^2^(*df* = 1) = 10.82, *p* < 0.05.

#### 3.6.1. Wall Post Communication Strategy and Medication Management 

Medication management was the most common self-management topic included within wall posts (48/180, 26.7%) (Table 5). Medication management posts were less likely to include a self-disclosure (40.0%), as compared to posts that did not (16.0%), *χ*^2^(df = 1) = 13.09, *p* < 0.001. A lower proportion of medication management posts referred group members to an external web source (13.2%, as compared to the posts that did include this service (32.3%), *χ*^2^(*df* = 1) = 6.96, *p* < 0.05. Medication management posts were also more likely to request information from other online users, *χ*^2^(*df* = 1) = 16.17, *p* < 0.001. 

#### 3.6.2. Wall Post Communication Strategy and Hospitalizations/Doctor Visits

Hospitalizations/doctor visits were the second most common self-management topic included in group wall posts (28/180, 15.6%) (Table 6). Posts highlighting a hospitalization or doctor’s visit were more likely to include self-disclosures (33.8%) than to withhold personal information (1.0%), *χ*^2^(*df* = 1) = 36.29, *p* < 0.001. None of the wall posts that addressed hospitalizations/doctor visits referred group members to an external web source. Finally, posts that did not address hospitalizations/doctor visits (97.8%) were far more likely to provide information as compared to posts that did not (2.2%), *χ*^2^(*df* = 1) = 8.12, *p* < 0.05.

### 3.7. Engagement with COPD Wall Posts according to Communication Strategy 

Results from the Mann-Whitney *U* Test showed that the number of likes on COPD group wall posts was significantly greater for posts that did not provide information about COPD (*Mdn* = 4) compared to posts that did provide information about COPD (*Mdn* = 2), *U* = 1996.00, *p* = *0*.001. In addition, the number of comments on group wall posts was significantly greater for posts that did not provide information about COPD (*Mdn* = 7) as compared to posts that did (*Mdn* = 1), *U* = 1256.5, *p* < 0.001. The number of likes for wall posts requesting information about COPD was significantly greater for posts that did not request information about COPD (*Mdn* = 4) as compared to posts that did (*Mdn* = 3), *U* = 2843.5, *p* = *0*.004. In addition, the number of comments on wall post requests for information was significantly greater for posts that did request information about COPD (*Mdn* = 7) as compared to posts that did not (*Mdn* = 2), *U* = 8813.5, *p* < 0.001. The number of likes was significantly greater for wall posts that demonstrated peer-to-peer (social) support (*Mdn* = 9.5) as compared to posts that did not demonstrate social support (*Mdn* = 3), *U* = 985.50, *p* < 0.001. 

## 4. Discussion

The current study explored online content and communication strategies used among members of COPD Facebook groups. This content analysis investigated how wall post communication strategies varied based on the presence of self-management topics in wall posts, specifically medication management and hospitalizations/doctor visits. The study also noted variability in engagement metrics according to the communication strategies used by members. To our knowledge, this is the first study to assess the content and group member communication strategies used in Facebook groups related to COPD. 

COPD Facebook groups were intended to exchange social support, rather than for the purposes of awareness building or fundraising. This finding is inconsistent with prior research examining the purpose of chronic disease self-management groups on social media. Bender and colleagues [37], for example, found that most Facebook groups related to breast cancer were created for awareness and fundraising purposes. Further, Greene and colleagues [43] reported that sharing information was the predominant communication strategy on diabetes-related social media communities. The differential prevalence of communication strategies used across chronic disease online forums highlights the value of developing and sustaining disease-specific, rather than generic chronic disease, communities. Future research should explore the discrete types of social support (emotional, appraisal, information, instrumental) provided within online communities, such as Facebook Groups, dedicated to COPD. 

The most common communication strategy used in COPD-related Facebook wall posts was self-disclosure (i.e., information was revealed about personal experiences with COPD), followed by making referrals to external web sources for additional information on topics of interest. Further examination of posts found that personal self-disclosures about the COPD experience and requests for information were more likely to be used as communication strategies in self-management posts (e.g., medication management, hospitalizations/doctor’s visits). As such, COPD Facebook Group members are exhibiting a form of communication competence, by contextualizing their question with information about the COPD experience, presumably to increase the relevance and accuracy of responses from other members. The high reliance on self-disclosures in this context may help to explain the substantial disparity between the number of members in closed (*n* = 84,684) versus public COPD Facebook groups (*n* = 2398), as social support and self-disclosures usually include more sensitive information being shared, and thus are more likely to occur in private groups. Research is needed to understand the social circumstances under which patients with COPD are more or less likely to disclose personal information on the Internet. 

Results of this study also demonstrate that referring patients to an external web source was a common communication strategy across the groups; however, referrals were less common in self-management posts. External web sources, especially those developed by reputable health-related organizations and agencies (e.g., NIH, CDC, WHO), provide evidence-based health information; therefore, information being shared within COPD Facebook groups may not be valid or accurate based on the latest scientific research. This is particularly concerning given that patients with COPD are only moderately confident when making health decisions based on information gained from the Internet [20]. Patients with COPD also report a low degree of health literacy [50]. To optimize content evaluation and site navigation, plain language standards recommend against directing an online user to an outside website as a universal health literacy precaution [51]. It is promising that self-management posts did not include these external website referrals; however, their prevalence across COPD Facebook Groups brings attention for the need to determine what exactly members are being directed toward outside of the online community. 

In this study, COPD Facebook Group members requested information about self-management. Users who addressed medication management were more likely to request health information. Due to the importance of medication adherence in COPD, there may be a need for physicians, pharmacists, and patient/health educators to collaborate on novel ways to support patient use of medications via COPD Facebook Groups. Posts that addressed medication management specifically rarely included member referrals to reliable sources of health information. This was likely due to the “closed” (private) nature of the vast majority of COPD Facebook groups identified in this cross-sectional study. While information sharing seems to occur primarily within the “group walls”, which may limit the dissemination of inaccurate information, the restricted nature of resource sharing limits what can be learned by Group members. Facebook group administrators should consider instituting policies that require moderators to post content from reputable governmental and non-profit sources based on the latest discussions and questions posted by group members. 

Discussions around hospitalizations or doctor visits were the second most common self-management topic discussed within group wall posts. Wall posts that included mentions of hospitalizations or doctor visits were more likely to include personal self-disclosures about the COPD experience, but they were less likely to include informational resources or provide external website resources. In other words, these posts were generally intended as a personal recount of the hospitalization or doctor visit experience. Despite the health communication potential of social media platforms such as Facebook groups, there may be a need for health care providers to also direct patients with COPD to their personal electronic health record to facilitate meaningful information exchange [52]. Facebook group administrators should configure processes that will alert group members that certain self-management topics are best broached within primary care through private patient-provider communication channels.

COPD Facebook Groups are very purposeful; they provide support from an emotional and informational perspective. While overall member engagement with wall posts was quite low, supportive posts, even those that did not provide any additional health information, were more often “liked” than informative posts that lacked any encouraging messages. Likewise, posts whose initiator requested information about COPD from other members were met with a greater number of comments from the community. The few members “engaged” with wall post content seemed to gravitate to posts without self-management information about COPD, which insinuates that “information heavy” posts should be avoided in favor of encouragement and motivational support about how patients can live their best life with COPD. Future primary research with actual patients who live with COPD should seek to confirm or disconfirm patient preferences for using social media platforms such as Facebook groups. 

### Limitations 

This study had several limitations. First, this study only included COPD Facebook groups that posted content in English. “Secret” Facebook groups, where users cannot view group content unless invited by an administrator or current member, were excluded from this study. The increased privacy of these groups could have resulted in different communication strategies and discussion of different self-management topics. In addition, this study was cross-sectional in nature, which could have resulted in missing seasonal effects of COPD self-management covered within Facebook Groups. For example, collecting information during fall or winter may have resulted in more discussion about flu/pneumococcal vaccinations, which are strongly recommended for patients with COPD. Also, the group search was limited to the terms of “COPD”, “emphysema”, and “chronic bronchitis”. Therefore, the search may have excluded COPD Facebook groups that did not include these specific search terms in the group title. Furthermore, rather than identifying self-management topics through a qualitative approach, this study adopted pre-defined codes to identify and categorize wall post content. This non-grounded-theory approach did not account for the wide range of topics that could potentially be discussed within COPD Facebook Groups. Use of theory-based text mining or machine learning algorithms may help identify the broad array of communication patterns that may occur on group walls. 

## 5. Conclusions

Researchers have stated the need for further research regarding peer support groups on Facebook and their impact on chronic disease self-management [53]. In the current study, results show that COPD Facebook group members are utilizing Facebook groups to share their experience managing their condition. The findings of this study support several important implications for practice among health education specialists and health care providers; including, the potential of COPD Facebook groups for establishing social support networks among patients living with COPD, as the groups were found to primarily serve that purpose. COPD Facebook Groups are very purposeful from an emotional and informational support perspective; however, in the context of the CSM [40], understanding more about the interactions among users on the COPD Facebook Groups could potentially help practitioners understand patient processes involved in managing illness threats such as dyspnea triggers. Additional research with patients living with COPD should evaluate the dynamics of these behavioral processes underlying patient motivations and uses of social media sites for disease management and emotional support.

In addition, given most of the communication on the Facebook wall posts involved patient self-disclosures, patient privacy protections need to be considered by practitioners who use Facebook groups as a platform for self-management education. In a similar vein, as COPD Facebook Group members request information about self-management, specifically medication management, it is important that physicians, pharmacists, and patient health education specialists work collaboratively to best use this platform to support the medication compliance and adherence needs that surfaced. While many COPD Facebook group members request information about self-management, there is a need for more informational and motivational support on these platforms to adequately support members in addressing their needs. Further research should explore the quality and safety of self-management information exchanged among patients with COPD on this popular social media platform. In addition, further research is needed to explore additional online user behaviors that quantify how COPD patients are utilizing information found within chronic disease self-management Facebook Groups, including those that are private. Patient dependence on social media tools for self-management, as opposed to reliance on their health care provider(s) for support, should also be explored further. This research will help produce a better understanding of unique patient preferences and motives for utilizing social media for COPD self-management.

## Figures and Tables

**Table 1:** Facebook group content analysis codes, definitions, and code sources.

Code	Definition	Code Source	Illness Self-Regulation Representation ^a^
***Group Purpose***			
Building Awareness/Sharing Information	Created to bring attention to the importance of COPD and share COPD-related information	Bender et al. [37]	Comorbid identities, causes, curability, consequences
Providing Support	Created to meet the emotional needs of COPD patients and their caregivers	Bender et al. [37]	Comorbid identities, causes, curability, consequences
Fundraising	Created to attract financial resources for COPD	Bender et al. [37]	N/A
Marketing/Promoting a Product/Service	Created to promote a COPD-related product or service	Bender et al. [37]	Curability
***Media Modality***			
Text	Text included in the post	Neiger et al. [42]	N/A
Video	Video included in the post	Neiger et al. [42]	N/A
Photo	Photo included in the post	Neiger et al. [42]	N/A
Infographic	Infographic included in the post	Neiger et al. [42]	N/A
***Communication Strategies***			
Self-Disclosures	Information was self-disclosed about personal experiences with COPD	Lerman et al. [38]	Comorbid identities, causes, curability, consequences
Referrals	Providing information about COPD through links to external websites, pages, groups, or documents	Lerman et al. [38]	Comorbid identities, causes, curability, consequences
Information Providing	COPD-related information was shared in the post	Greene et al. [43]	Comorbid identities, causes, curability, consequences
Information Requesting	COPD-related queries were posed to the group	Greene et al. [43]	Comorbid identities, causes, curability, consequences
Demonstrating Support	Emotional support was provided to the group	Greene et al. [43]	Comorbid identities, causes, curability, consequences
Product/Service Promotion	A product or service was promoted	Greene et al. [43]	Curability
***Self-Management Content Area***			
Mental Health	Mentions mental health issues such as depression, anxiety, etc.	CDC [44]	Comorbid identities, Consequences
Medications	‘Mentions medications such as antibiotics, inhalers, etc.	CDC [44]	Curability
Hospitalizations/Doctor Visits	Mentions hospitalization or doctors visit related to COPD	GOLD [45]	Consequences
Asthma	Mentions asthma specifically	GOLD [45]	Comorbid identities
Smoking	Mentions smoking such as cigarette smoking, vaping, e-cigarette use, etc.	GOLD [45]	Causes
Breathing Techniques	Mentions specific breathing techniques to cope with exacerbations	CDC [44]	Curability
Nutrition	Mentions nutrition such as recipes and health eating habits	CDC [44]	Curability
Physical Activity	Mentions physical activity such as walking, stair climbing, etc.	CDC [44]	Curability, Consequences
***Engagement***			
Like	Number of times members reacted to the post by pressing the thumbs up icon	Neiger et al. [42]	N/A
Love	Number of times members reacted to the post by pressing the heart emoji	Neiger et al. [42]	N/A
Sad	Number of times members reacted to the post by pressing the sad emoji	Neiger et al. [42]	N/A
Angry	Number of times members reacted to the post by pressing the angry emoji	Neiger et al. [42]	N/A
Laugh	Number of times members reacted to the post by pressing the haha emoji	Neiger et al. [42]	N/A
Surprised	Number of times members reacted to the post by pressing the wow emoji	Neiger et al. [42]	N/A
Comment	Number of times members reacted to the post by replying with text, gif, etc.	Neiger et al. [42]	N/A
Share	Number of times members reacted to a post by sharing it	Neiger et al. [42]	N/A

^a^ Illness self-regulation representations were coded based on information from Leventhal et al. [40] and Hale et al. [46].

**Table 2:** Intercoder reliability scores for self-management content areas and characteristics of Chronic Obstructive Pulmonary Disease (COPD) group wall posts (*n* = 40).

**Self-Management Content Area**	**Cohen’s Kappa**
Mental Health	>0.99
Medications	>0.99
Hospitalizations/Doctor Visits	0.91
Asthma	>0.99
Smoking	>0.99
Breathing Techniques	0.66
Nutrition	>0.99
Physical Activity	0.66
*Mean*	0.90
*SD*	0.15
**Wall Post Characteristics**	**Cohen’s Kappa**
Post Included Text	0.66
Post Included Video	>0.99
Post Included Photo	0.87
Post Included Infographic	>0.99
Information Providing	>0.99
Information Requesting	0.95
Demonstrating Support	0.75
Personal Self-Disclosure	0.95
Referral to Other Resources	>0.99
Product/Service Promotion	>0.99
*Mean*	0.91
*SD*	0.12

**Table 3:** General characteristics and membership in 26 COPD Facebook groups as of March 2019.

Group Name	Privacy Setting	Group Purpose	Number of Members	Number of New Members in Past 30 Days	Number of Posts in Past 30 Days	Months in Operation
Ultimate Pulmonary Wellness: COPD, PF, Pulmonary Hypertension and Others	Closed	Providing Support	12,337	245	632	36
COPD Warriors Hope, Support, Love & Laughter	Closed	Providing Support	10,451	692	2764	60
COPD/Emphysema/Pulmonary Disease- I have COPD- COPD does not have me!	Closed	Building Awareness/Sharing Information	7952	0	367	60
COPD- GET EDUCATED!	Closed	Providing Support	6837	0	277	24
(COPD) Emphysema/Chronic Bronchitis Support Group	Closed	Providing Support	5537	0	67	120
COPD Information and Support	Closed	Building Awareness/Sharing Information	5028	294	1087	48
Let’s Talk COPD Support Group	Closed	Providing Support	4139	213	672	24
Support Group for People with COPD, Emphysema, Asthma, Bronchitis	Closed	Providing Support	3938	192	233	84
Emphysema	Closed	Providing Support	3794	84	243	96
COPD Xplained- USA	Closed	Building Awareness/Sharing Information	3748	90	291	108
COPD Support Group	Closed	Providing Support	3049	229	551	36
COPD/ A WARM LOVING PLACE TO HANG OUT	Closed	Providing Support	3027	97	1998	48
COPD Breathing Buddies	Closed	Providing Support	2776	0	660	60
Lift Up- COPD Support Group	Closed	Providing Support	2438	282	381	12
COPD- New Treatments and Advice	Closed	Building Awareness/Sharing Information	2238	0	34	48
COPD/COAD Support	Closed	Building Awareness/Sharing Information	2223	0	125	96
COPD/ALPHA 1	Public	Providing Support	1273	4	59	60
COPD- A BREATH OF FRESH AIR	Closed	Providing Support	974	0	272	48
COPD-Emphysema-Chronic Bronchitis	Closed	Providing Support	886	0	6	96
COPD Tackling it Together	Closed	Providing Support	868	0	103	132
COPD Service	Public	Providing Support	830	0	0	60
COPD CRAZINESS AND SUPPORT	Closed	Providing Support	810	0	301	120
Chronic Obstructive Pulmonary Disease	Closed	Providing Support	781	0	7	96
C.O.P.D Warriors	Closed	Building Awareness/Sharing Information	700	0	343	24
Support & Awareness for COPD, Emphysema and Chronic Bronchitis	Public	Building Awareness/Sharing Information	295	0	6	132
Chronic Bronchitis Support & Awareness	Closed	Providing Support	153	0	0	12

**Table 4:** Frequency of wall post content (*n* = 180) of 26 COPD Facebook Groups according to whether or not wall post addressed any self-management topics.

Wall Post Communication Strategies	Posts Addressing COPD Self-Management *n* (%)	Posts Not Addressing COPD Self-Management *n* (%)	*p* Value
**Self-Disclosure**			
Yes (*n* = 80)	50 (62.5) **	30 (37.5)	0.0001
No (*n* = 100)	28 (28)	72 (72)	
**Referral to External Information Source**			
Yes (*n* = 53)	13 (24.5) *	40 (75.5)	0.001
No (*n* = 127)	65 (51.2)	62 (48.8)	
**Information Provided**			
Yes (*n* = 45)	19 (42.2)	26 (57.8)	0.862
No (*n* = 135)	59 (43.7)	76 (56.3)	
**Information Requested (i.e., Asks a Question)**			
Yes (*n* = 69)	42 (60.9) **	27 (39.1)	0.0001
No (*n* = 111)	36 (32.4)	75 (67.6)	
**Offers Support**			
Yes (*n* = 26)	2 (7.7) **	24 (92.3)	0.0001
No (*n* = 154)	76 (49.4)	78 (50.6)	
**Product/Service Promotion**			
Yes (*n* = 5)	3 (60)	2 (40)	0.446
No (*n* = 175)	75 (42.9)	100 (57.1)	

* *p* < 0.05, ** *p* < 0.001.

**Table 5:** Frequency of communication strategies used in COPD Facebook group wall posts according to whether or not post addressed medication management (*n* = 48).

Communication Strategy	Post Addressed Medication Management *n* (%)	Post Did Not Address Medication Management *n* (%)	*p* Value
**Self-Disclosure**			
Yes (*n* = 80)	32 (40) **	48 (60)	0.0001
No (*n* = 100)	16 (16)	84 (84)	
**Referral to External Information Source**			
Yes (*n* = 53)	7 (13.2) *	46 (86.8)	0.008
No (*n* = 127)	41 (32.3)	86 (67.7)	
**Information Provided**			
Yes (*n* = 45)	10 (22.2)	35 (77.8)	0.436
No (*n* = 135)	38 (28.1)	97 (71.9)	
**Information Requested (i.e., Asks a Question)**			
Yes (*n* = 69)	30 (43.5) **	39 (56.5)	0.0001
No (*n* = 111)	18 (16.2)	93 (83.8)	
**Offers Support**			
Yes (*n* = 26)	0 (0) *	26 (100)	0.001
No (*n* = 154)	48 (31.2)	106 (68.8)	
**Product/Service Promotion**			
Yes (*n* = 5)	1 (20)	4 (80)	0.732
No (*n* = 175)	47 (26.9)	128 (73.1)	

* *p* < 0.05, ** *p* < 0.001.

**Table 6:** Frequency of communication strategies used in COPD Facebook group wall posts based on whether or not post addressed hospitalizations/doctor visits (*n* = 28).

Type of Communication Strategy	Post Addressed Hospitalizations/Doctor Visits *n* (%)	Post Did Not Address Hospitalizations/Doctor Visits *n* (%)	*p* Value
**Self-Disclosure**			
Yes (*n* = 80)	27 (33.8) **	53 (66.2)	0.0001
No (*n* = 100)	1 (1)	99 (99)	
**Referral to External Information Source**			
Yes (*n* = 53)	0 (0) **	53 (100)	0.0001
No (*n* = 127)	28 (22.0)	99 (78.0)	
**Information Provided**			
Yes (*n* = 45)	1 (2.2) *	44 (97.8)	0.004
No (*n* = 135)	27 (20)	108 (80)	
**Information Requested (i.e., Asks a Question)**			
Yes (*n* = 69)	15 (21.7)	54 (78.3)	0.071
No (*n* = 111)	13 (11.7)	98 (88.3)	
**Offers Support**			
Yes (*n* = 26)	2 (7.7)	24 (92.3)	0.232
No (*n* = 154)	26 (16.9)	128 (83.1)	
**Product/Service Promotion**			
Yes (*n* = 5)	0 (0)	5 (100)	0.330
No (*n* = 175)	28 (16)	147 (84)	

* *p* < *0*.05, ** *p* < *0*.001.
